# Characterization and Identification of a Ripening-Related Gene *AaPG18* in *Actinidia arguta*

**DOI:** 10.3390/ijms23052597

**Published:** 2022-02-26

**Authors:** Yukuo Li, Hailei Huang, Muhammad Abid, Hong Gu, Jinbao Fang, Zhongping Cheng, Xiujuan Qi

**Affiliations:** 1 Zhengzhou Fruit Research Institute, Chinese Academy of Agricultural Sciences, Zhengzhou 450009, China; liyukuo@caas.cn (Y.L.); 82101195082@caas.cn (H.H.); guhong@caas.cn (H.G.); fangjinbao@caas.cn (J.F.); 2 Lushan Botanical Garden, Chinese Academy of Sciences, Jiujiang 332900, China; muhammadabid@lsbg.cn; 3 Wuhan Botanical Garden, Chinese Academy of Sciences, Wuhan 430074, China

**Keywords:** *Actinidia arguta*, fruit ripening, gene expression, *AaPG18*, gene function

## Abstract

*Actinidia arguta* (*A. arguta*) is a kind of climacteric fruit that quickly softens and limits fruit shelf-life and commercial value. Therefore, it is of great significance to develop kiwifruit genotypes with an extended shelf-life of fruit. However, the ripening and softening mechanisms remain unclear in *A. arguta*. Here, we demonstrated that a key polygalacturonase (PG)-encoding gene *AaPG18* was involved in *A. arguta* ripening through the degradation of the cell wall. Fruits were harvested at three developmental stages (S1, S2, and S3) for high-throughput transcriptome sequencing, based on which two candidate transcripts *c109562_g1* and *c111961_g1* were screened. The genome-wide identification of the PG gene family assigned *c109562_g1* and *c111961_g1* to correspond to *AaPG4* and *AaPG18*, respectively. The expression profiles of candidate genes at six preharvest stages of fruit showed significantly higher expression levels of *AaPG18* than *AaPG4*, indicating *AaPG18* might be a key gene during fruit ripening processes. The subcellular localization displayed *AaPG18* was located at the cytoplasmic membrane. The transient overexpression of *AaPG18* in strawberry and the following morphological observation suggested *AaPG18* played a key role in maintaining the stability of cell morphology. The homologous transient transformation in *A. arguta* “RB-4” proved the crucial function of *AaPG18* in fruit ripening processes by causing the rapid redness of the fruit, which was an indicator of fruit maturity. All in all, our results identified *AaPG18* as a key candidate gene involved in cell wall degeneration, which provides a basis for the subsequent exploration of the molecular mechanisms underlying the ripening and softening of *A. arguta* fruit.

## 1. Introduction

Kiwifruit possesses rich germplasm resources, including 54 species and 21 varieties [1]. For decades, the international market was mainly occupied by *Actinidia chinensis* Planchon (*A. chinensis*) and *Actinidia chinensis var. deliciosa* A. Chevalier (*A. chinensis* var. *deliciosa*). *Actinidia arguta* (Siebold and Zuccarini) Planchon ex Miquel *(A. arguta*), a miniature kiwifruit, gradually developed into the second largest species to be cultivated worldwide [2]. However, *A. arguta* fruit at maturity is highly prone to softening, resulting in a shortened shelf-life. Therefore, it is necessary to explore the molecular mechanism underlying fruit ripening to provide a basis for the breeding of new cultivars with an extended shelf-life.

Fruit ripening is a continuous and dynamic process, accompanied by a series of physiological and biochemical changes including fruit color, fruit texture, and fruit hardness [3,4]. Cell wall metabolism is the key stimulus causing the change in fruit texture. Enzymes encoding genes for cell wall degradation such as polygalacturonases (*PG*s) and pectate lyases (*PEL*s) play vital roles in the softening process through the disassembly and dissolution of cell wall components [5,6]. Previous studies have confirmed that the biotechnological silencing or knockout of these ripening-specific expressed genes would delay or reduce fruit softening, suggesting that these genes play an indispensable role in fruit ripening [7,8,9,10]. The apple *MdPG1* was proved to be a softening-related gene that is induced by ethylene and cold treatment during fruit ripening and is specifically activated by both ethylene-insensitive3-like (EIL) and cold binding factor (CBF) transcription factors (TFs) [11]. Likewise, *FaPG1* was demonstrated to be a key *PG* gene associated with fruit softening in strawberries by the antisense-mediated reduced expression of *FaPG1*, which attenuates fruit softening and extends the shelf life [12,13]. Although a few studies about *PGs* involved in fruit ripening have been investigated in kiwifruit, most of them focused on the expression of *PGs* and physiological changes, such as the solubilization of pectic backbone in the cell wall and the appearance of loose cytoskeleton [14,15]. So far, the knowledge about molecular mechanism underlying kiwifruit ripening has been scarce and demands extensive exploration. *A. arguta* is a kind of newly developing kiwifruit species that is typically climacteric with rapid softening after fruit maturity, which greatly limits its commercial value. Previous studies revealed various TFs involved in the ripening processes of fruits using the comparative transcriptome analysis of *A. arguta* at the commercial harvest stage and six days after harvest [16]. However, the key candidate genes, especially the cell wall-degrading genes, have not been studied well in kiwifruit for ripening mechanism.

In this study, fruits at six ripening stages were used to determine the physiological indexes. The candidate genes were screened by RNA-seq. The *PG* family members were identified at the genome-wide level. The key gene *AaPG18* was cloned and characterized for fruit softening by a series of experiments including homologous cloning, heterologous transformation, and transient overexpression. Our findings provide a molecular explanation for *A. arguta* fruit ripening and softening.

## 2. Results

### 2.1. Physiological Changes in Fruit at Different Preharvest Stages

**Figure 1:**
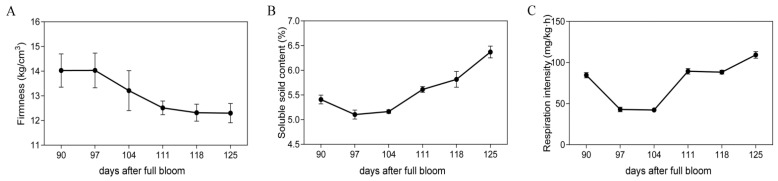
Physiological indexes investigation. (**A**) Firmness values of fresh fruit flesh at six preharvest stages. (**B**) Soluble solid contents of fresh fruit flesh at six preharvest stages. (**C**) Respiration intensities of fresh fruit flesh at six preharvest stages. At least three readings were used to plot one point. The data represent the error bars ± SE (Standard Error) of three biological replicates.

To investigate the ripening status of “TY” fruits, samples from six preharvest stages were assessed for fruit firmness, soluble solid content, and respiration intensity. The firmness of the fruits gradually decreased with ripening processes (Figure 1A), while the soluble solid content presented an upward trend (Figure 1B). In contrast, the respiration intensity showed a zigzag trend that initially decreased and then increased (Figure 1C). The changes in the above-mentioned indexes might be implicated in the dramatic changes of fruit during ripening processes.

### 2.2. Screening of Candidate Genes Based on Transcriptome Analysis

**Figure 2:**
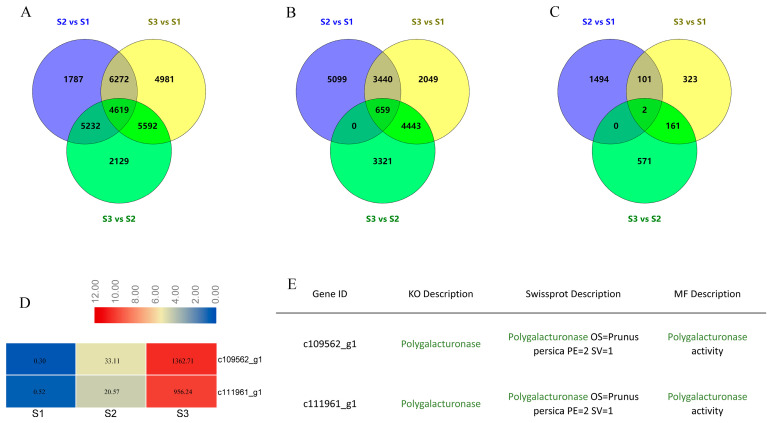
The Venn diagrams of differentially expressed genes (DEGs) and the expression levels of transcripts. (**A**) Venn diagrams of all DEGs among three comparisons including S2 vs. S1, S3 vs. S1, and S3 vs. S2. (**B**) Venn diagrams of upregulated DEGs among three comparisons including S2 vs. S1, S3 vs. S1, and S3 vs. S2. (**C**) Venn diagrams of upregulated DEGs with a 5-fold change among three comparisons including S2 vs. S1, S3 vs. S1, and S3 vs. S2. (**D**) Expression levels of two key transcripts including c109562_g1 and c111961_g1. (**E**) Annotations of c109562_g1 and c111961_g1. The green font indicates c109562_g1 and c111961_g1 were both annotated as polygalacturonase in three known online databases including KO, Swissprot, and MF.

The transcriptome analysis of fruit flesh samples at three critical stages showed that large numbers of genes were strongly expressed during fruit ripening process. Three comparisons, S2 vs. S1, S3 vs. S1, and S3 vs. S2, were used for finding candidate genes. A total of 4619 differentially expressed genes (DEGs) were co-expressed among these three comparisons (Figure 2A, Table S1), of which 659 DEGs were upregulated at each consecutive time point (Figure 2B). The most significant DEGs during fruit ripening were screened by setting cutoff values for transcripts at a five-fold change as the screening threshold. Finally, two transcripts, c109562_g1 and c111961_g1, were identified as potential candidate genes (Figure 2C). The expression levels of these two transcripts were significantly higher in S3 than those in S1 (Figure 2D). Both transcripts were annotated as PG on different online databases (KO, Swissprota, and MF) (Figure 2E). 

### 2.3. Identification and Classification of the PG Family in Kiwifruit

**Figure 3:**
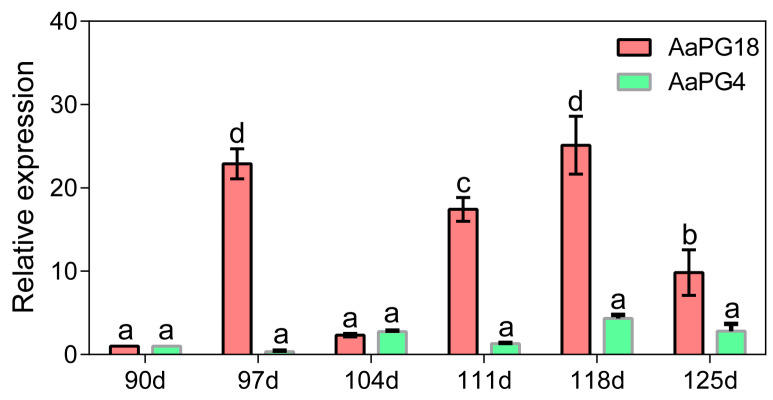
The expression levels of *AaPG4* and *AaPG18* in fresh fruit flesh at six preharvest stages. Kiwifruit *β-actin* was selected as an internal control gene during qPCR. Values are presented as means ± SD for three replicates. The statistical significance of mean values is indicted by different letters.

A genome-wide identification of *the PG family* was carried out to find the close relation of c109562_g1 and c111961_g1 with other family members. A total of 52 PG family members were excavated from kiwifruit “R5” genome. The chromosomal distribution showed these 52 *PGs* were irregularly located on 20 different chromosomes including chromosomes 02, 03, 05, 06, 07, 08, 11, 12, 13, 14, 15, 16, 17, 18, 19, 20, 22, 23, 28, and 29 and five members were distributed in chromosomes 12, 17, and 29, while the rest of the members were found on chromosomes 11, 13, 14, 16, 20, 22, and 23 (Figure S1). We conducted structural analysis, phylogenetic evolution, domain organization, and conserved motif analysis to find out the closely related PG family members of c109562_g1 and c111961_g1. Fifty-two *PGs* were further divided into six sub-families, namely A, B, C, D, E, and F. Through the sequencing and homology comparisons, we found that c109562_g1 and c111961_g1 corresponded to *AaPG4* and *AaPG18*, respectively. Additionally, AaPG4 and AaPG18 were clustered into sub-family A (Figure S2). The expression levels of *AaPG4* and *AaPG18* in samples at six preharvest stages were assessed by qRT-PCR. The results showed that both genes were expressed during fruit development. Except for the low expression of *AaPG18* at 104 d, that should be a stage at which the next maturity was prepared, following which the transcription level of *AaPG18* presented an enhancing trend at 111, 118, and 125d. In general, it was noteworthy that the transcription level of *AaPG18* was significantly higher than that of *AaPG4* during the whole process of fruit maturity, indicating that *AaPG18* might play an important role during fruit ripening (Figure 3).

### 2.4. A Membrane-Localizated AaPG18 Protein

**Figure 4:**
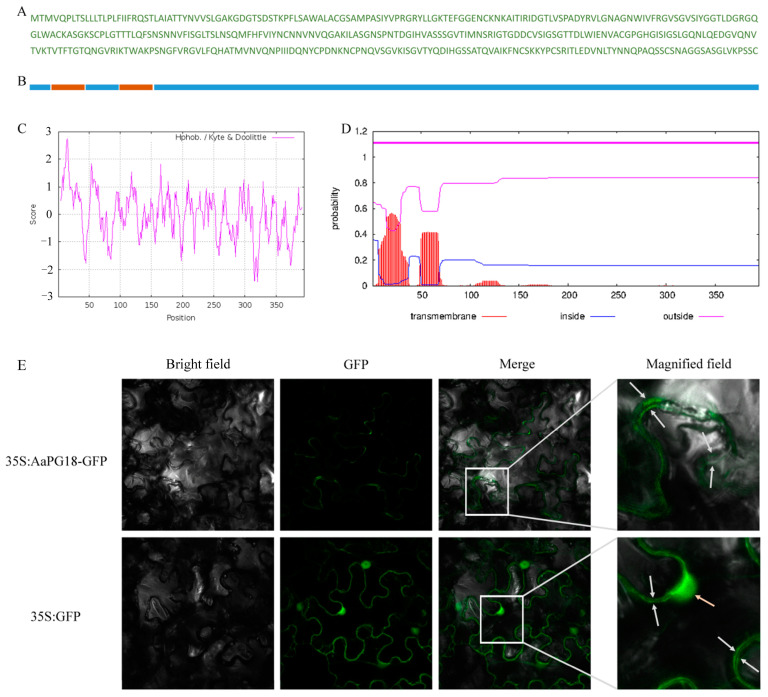
Protein structure analysis and subcellular localization of AaPG18. (**A**) Deduced protein sequence of AaPG18 with 395 amino acids. (**B**) Schematic representation of AaPG18 protein structure. Two possible N-terminus transmembrane domains (orange boxes) were included. (**C**) Hydropathic profile of AaPG18 protein using the predicting tool ProtScale (https://web.expasy.org/protscale/, accessed on 25 November 2021). (**D**) Prediction of transmembrane structure using TMHMM-2.0 (http://www.cbs.dtu.dk/services/TMHMM/, accessed on 25 November 2021). (**E**) Subcellular localization of AaPG18 in Nicotiana benthamiana (N. benthamiana) leaves. Cells expressing AaPG18–GFP fusion gene exhibited GFP fluorescence signals which were distributed in the cytoplasmic membrane. Cells expressing an empty vector only with a GFP tag showed GFP fluorescence signals at the nucleus and membrane. Three observed fields including brightness, GFP, and mergence were captured. The magnified field showed GFP signals in different parts of cells. White and orange arrows indicate the presence of GFP signals in the cell membrane and nucleus, respectively.

The subcellular localization of AaPG18 protein in the plant was predicted by an online tool PSORT (https://www.psort.org/psortb/index.html, accessed on 25 November 2021). The localization scores (cytoplasmic: 0.32; cytoplasmic membrane: 9.55; cell wall: 0.12; extracellular: 0.01) predicted its presence in the cytoplasmic membrane. To further determine this predicted result, we have analyzed the deduced amino acid sequence of PG18 in two different ways to find out the membrane-spanning domain. As expected, there were two N-terminal membrane-spanning domains in the deduced AaPG18 protein (Figure 4A–D). To gain the direct evidence of its localization in plant cells, the constructs for subcellular localization were introduced into *N. benthamiana* leaves. The GFP fluorescence signal was detected only in the cytoplasmic membrane of leaves injected with 35S:AaPG18-GFP, while it was observed in both the nuclear and membrane of leaves injected with the control (35S:GFP), which confirmed our initial prediction that AaPG18 might be a membrane-localized protein and worked in the outer layer of the cell (Figure 4E). 

### 2.5. Transient Overexpression of AaPG18 in Strawberry

**Figure 5:**
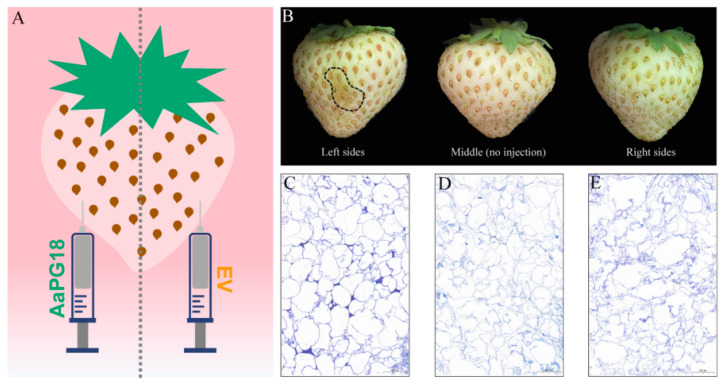
Transient overexpression in fruits of strawberry “snow”. (**A**) Diagrammatic sketch of the transient injection on the left and right sides of the same fruit of strawberry. (**B**) Phenotype observation of strawberry three days after injection. (**C**) Morphological observation of the cell section of strawberry fruit infiltrated with pBI121–AaPG18. (**D**) Morphological observation of the cell section of strawberry fruit without any injection. (**E**) Morphological observation of the cell section of strawberry fruit infiltrated with pBI121 (empty vector) as the control. The scale bar: 200 μm.

To explore the gene function of AaPG18, the transient overexpression was carried out in strawberry cultivar “snow” fruits using the method of the single fruit control (Figure 5A). The side injected with pBI121–AaPG18 showed an obvious grayish brown color in the infiltrated areas, while nothing happened on the other side of fruit injected with only pBI121 or no injection at all (Figure 5B). In order to find the reason behind this differential phenomenon at the cellular level, the infiltrated tissue sections were stained with toluidine blue O. The cross-section of the fruit side overexpression of AaPG18 presented the regular and flat status of cells (Figure 5C), while the cells from the fruit section infiltrated with the control or not infiltrated at all were irregular and loose in shape (Figure 5D,E), which indicated that the AaPG18 played a key role in cell wall stability and it helped the cell to maintain its morphology.

### 2.6. Homologous Transient Transformation of AaPG18 in “RB-4”

**Figure 6:**
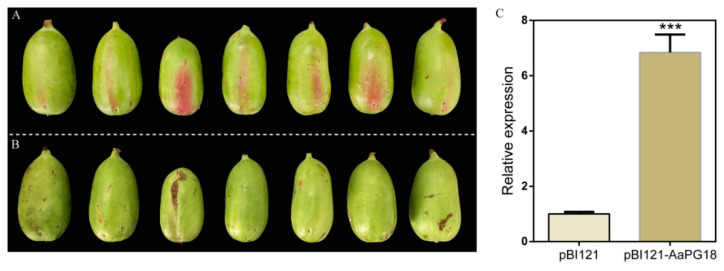
Homologous overexpression in fruits of *A. arguta* “RB-4”. (**A**) Phenotype of the side infiltrated with pBI121–AaPG18 presenting an obvious red color. (**B**) Phenotype of the side infiltrated with pBI121 presenting no change. (**C**) Expression level of *AaPG18* in “RB-4” fruits infiltrated with pBI121–AaPG18 and pBI121. Similar to strawberry fruit, the left and right sides of one fruit were injected with pBI121–AaPG18 and pBI121, respectively. Values are presented as means ± SD for three replicates. Statistical significance: *** p < 0.001.

The homologous transformation is considered among the best ways to investigate gene function. To understand the roles of AaPG18 in kiwifruit, the transient overexpression was conducted in *A. arguta* “RB-4” by using the signal fruit control method. The fruit side injected with pBI121–AaPG18 turned red (Figure 6A), while nothing appeared on the side infiltrated with the empty vector (Figure 6B). The appearance of red color in “RB-4” overexpressing AaPG18 indicated its involvement in fruit maturity. Therefore, these obvious phenotypic results confirmed the key role of AaPG18 in fruit ripening and softening. The qPCR resulted showed a significantly higher expression level of AaPG18 on the fruit side infiltrated with pBI121–AaPG18 than on the opposite side infiltrated with pBI121 (Figure 6C).

## 3. Discussion

The degradation of the cell wall is mainly responsible for the softening of kiwifruit fruit texture. Pectin is the major cell wall component that affects the cellular integrity and cell adhesion and thereby plays a key role in fruit softening [17]. PG has known to be the largest hydrolase family involved in pectin disassembly. Therefore, the identification of the PG family is of significant importance for studying molecular mechanisms underlying fruit ripening. Until recently, the genome-wide identification of the PG family has been conducted in various plants including *Solanum lycopersicum* [18], *Mangifera indica* [19], *Pyrus bretschneideri* [20], *Vitis vinifera* [21], and *Malus domestica* [22], aiming to discover the key *PG* genes involved in fruit ripening processes. Although several attempts have been made to explore *PG* genes in kiwifruit [23,24,25,26,27,28,29], the key *PG* members related to fruit ripening remain unclear. In the current study, we used fruit samples from different ripening stages for transcriptome sequencing and combined physiological indexes results to explore the key *AaPG18* gene in kiwifruit. Both the online prediction and the green fluorescence protein label test suggested that AaPG18 was a membrane-localized protein and had function in the outer layer of the cell. In order to obtain the direct evidence that *AaPG18* had an obvious effect on fruit ripening, we carried out the heterologous and homologous transient overexpression in strawberry “snow” and kiwifruit “RB-4”, respectively, by using the method of the strict single fruit control. As we expected, the overexpression of *AaPG18* accelerated strawberry-ripening processes evidently through phenotypic observations and the section cytology study (Figure 5). The most heartening thing for us was the significant changes in *A. arugta* “RB-4” fruit after the overexpression of *AaPG18*. The side of fruit infiltrated with pBI121–AaPG18 turned red rapidly within two days compared with the side infiltrated with pBI121 (control). The higher expression level of AaPG18 in fruit part overexpressing AaPG18 suggested its critical role in fruit softening and ripening by decomposing the pectin in fruit (Figure 6). Unfortunately, the verification of functional genes in fruit trees is extremely time-consuming. If the gene transformation experiment can be carried out successfully in own species, it will directly confirm the key role of the gene for a particular biological trait or the process of growth and development. Therefore, the method of the strict single fruit control in this study may provide a reference for the identification of functional genes in other species, but the specific details of this method applicable to specific species may need further modification or improvement.

Fruit ripening is an extremely complex process, which involves a series of dynamic changes in enzyme activity and deliberate the regulation of the differential expression of genes. Many genes involved in this process have been cloned and identified in different species. In addition to the PG genes mentioned above, many TFs can also participate in the regulation of fruit ripening and softening. Numerous TFs, such as MYB, bHLH, and NAC, have been identified as important regulators to control fruit ripening and softening. FaSPT (bHLH TF) and FaMADS9 (SEPALLATA1/2-like) were proposed to regulate fruit settings and ripening [30,31]. The NAC TFs family members involved in the ripening-associated process have also been found in *Prunus persica* [32], *Solanum lycopersicum* [33], and *Malus domestica* [34]. Some TFs and miRNA-TFs modules involved in fruit ripening have been recently characterized in kiwifruit [35,36,37,38], which provides a good basis and direction for future research on the regulation mechanism of kiwifruit ripening. In the current study, we did not mention ripening-related regulators, because our research was specifically focused on one key functional gene involved in cell wall degradation, an important phenomenon in fruit softening and ripening. Therefore, we will screen the upstream regulators of *AaPG18* to explore the regulatory network of ripening mechanisms in our subsequent study.

## 4. Materials and Methods

### 4.1. Fruits Preparation and RNA-Seq Process

*A. arguta* cultivar “Tianyuanhong” (“TY”) fruits were selected as experimental materials from National Horticulture Germplasm Resources Center-Kiwifruit Germplasm Repository in Zhengzhou Fruit Research Institute, the Chinese Academy of Agricultural Sciences located at Zhengzhou in Henan Province. The fresh fruits at six preharvest stages (90, 97, 104, 111, 118, and 125 days after full bloom) were sampled from three independent vines. We collected at least 20 fruits for each stage. In addition, the fruit flesh sampled at the early development stage (S1, 70 days after full bloom), the middle development stage (S2, 100 days after full bloom), and the fully ripened stage (S3, 130 days after full bloom) were used for high-throughput RNA sequencing. At least 10 fruits with good conditions from independent vines for one replicate were used for separating peels. The total RNA used for library construction was isolated from nine samples using the (cetyltrimethyl ammonium bromide (CTAB) method, after which RNA contamination and purity were detected by gel electrophoresis and a NanoDrop system (Thermo Fisher Scientific, Waltham, MA, USA), respectively. The qualified RNAs were constructed to form a cDNA library with a reasonable concentration greater than 2 nM. The Illumina HiSeq^TM^ (ILMN, lnc., San Diego, CA, USA) was selected as the sequencing platform to obtain raw sequences, which were cleaned by specific standards including adapter removing, low-quality read abandon, and unknown identity discard. The specific information of reads is presented in Table S2. In addition, the estimations and calculations of differential expression genes were using the RSEM and FPKM methods [39,40]. There were three biological replicates for samples at each developmental stage throughout the RNA-seq process.

### 4.2. Physiological Indexes Determination

The fruit flesh parts (unit: kg/cm^2^), after peeling, were selected to investigate firmness using a GY-3 typed fruit hardness tester with a measuring head size of 8 mm (Beijing THY Science & technology Co., Ltd., Beijing, China), and the performed parameters including the depth inserted, speed of probe, and trigger force were 9 mm, 3 mm/s, and 15 g, respectively. A batch of 10 fruits was tested at a time. The soluble solid content (unit: %) was measured by a VR-113 typed hand-held refractometer. The respiratory intensity (unit: CO_2_ mg/(kg·h)) was measured by standing lye absorption methods with slight modifications [41].

### 4.3. Genome Availability, Pfam Search, and Chromosomal Localization

To identify the PG members in kiwifruit, the “Red 5” (*Actinidia chinensis* var. *chinensis*) genome, was downloaded from the database Ensembl Plants (http://plants.ensembl.org/index.html, accessed on 25 November 2021) [42]. The PG members were searched by using the conserved domain of PG in the Pfam database Glyco_hydro_28 (PF00295) as a query in the Hmmsearch way. Afterwards, the specific information of chromosomal physical locations for the candidate *PG* family genes was obtained, and then the chromosomal localization chart was plotted using MapGene2Chrom Web v2 (http://mg2c.iask.in/mg2c_v1.1/, accessed on 25 November 2021) [43]. The chromosomal location information for the *PG* family members is presented in Table S3.

### 4.4. Sequence Alignment, Phylogenetic Tree Construction, and Gene Structure Model Analysis

A total of 52 *PG* genes were used for multiple sequence alignments, and then a phylogenetic tree was constructed with MEGA 4.1 version software (Mega Ltd., Auckland, New Zealand) by selecting the neighbor-joining method based on the protein sequence alignment results [44]. The gene structures including the presence of exon, intron, and 5′-/3′-untranslated regions (UTRs) were obtained and then used for producing the structure chart by the GSDS 2.0 online tool (http://gsds.cbi.pku.edu.cn/, accessed on 25 November 2021) [45]. The conserved motifs of PG proteins were predicted by MEME v5.1.1 (https://meme-suite.org/tools/meme/, accessed on 25 November 2021), in which the top number of motifs was set at three. The specific structural information for the *PG* family members is shown in Table S4.

### 4.5. Homologous Cloning and Vector Construction

The coding sequence of *AaPG18* was amplified from “TY” by RT-PCR (Reverse Transcription-Polymerase Chain Reaction) with a specific primer pair (forward 5′-ATGACAATGGTGCAACCACT-3′/reverse 5′-CTACAAGCAACTTGAGGGCTTAA-3′). The recovered PCR product after running agarose gel electrophoresis was transformed into E. coli (Escherichia coli) DH5α competent cells, after which the specific sequence was obtained by Sanger sequencing. Similarly, the coding region of *AaPG18* without a terminator was amplified by a specific primer pair (forward 5′-gagaacacgggggactctagaAAGATGACAATGGTGCAACCACT-3′/reverse 5′-gcccttgctcaccatggatccCAAGCAACTTGAGGGCTTAACTAA-3′) using the homologous recombination method. The accurate PCR product was recombined with the plant binary expression vector pBI121 to form CaMV 35S:AaPG18-GFP (Green Fluorescent Protein) (AaPG18–pBI121). An empty vector (only pBI121 with GFP) was used as the control.

### 4.6. Subcellular Localization in Nicotiana Benthamiana (N. Benthamiana)

Two constructs (AaPG18-pBI121 and pBI121) were introduced into *A. tumefaciens* (*Agrobacterium tumefaciens*) strain EHA105 using a freeze-thaw method. The bacterial cultures containing both constructs were separately re-suspended using an infiltration buffer containing 10 mM MgCl_2_, 10 mM MES, and 200 µM acetosyringone (AS) to OD_600_ (Optical Density 600) of 0.6–1.0 and placed at room temperature for 2 h before infiltration. The infiltration solution was injected into 5–6-week-old *N. benthamiana* leaves with a 1 mL needleless syringe. The infiltrated *N. benthamiana* plants were placed under dark conditions at room temperature for the first 24 h and then under 16 h light and 8 h dark for another 24 h. The GFP fluorescence was observed in the *N. benthamiana* leave cells after a plasmolysis treatment with a Leica TCS SP5 confocal laser scanning microscope (Leica Microsystems, Wetzlar, Germany).

### 4.7. Heterologous and Homologous Overexpression in Strawberry and Kiwifruit 

The constructed vector AaPG18–pBI121 was transiently overexpressed in strawberry and kiwifruit fruits by *A. tumefaciens*-mediated infiltration. The immature fruits of strawberry cultivar “snow” were infiltrated with a bacterial culture-containing construct by using a 1 mL needle syringe. An injection of the bacterial culture was performed on the left and right sides of each fruit using an infiltration buffer containing 10 mM MgCl_2_, 10 mM MES, and 150 µM AS with OD_600_ of 0.8. The phenotype changes were observed after three days of infiltration. Likewise, the immature *A. arguta* “RB-4” fruits (70–80 DAFB) were injected with pPB121–AaPG18 and pBI121 on the left and right sides of each fruit. The phenotype changes were observed two days after infiltration. We injected at least 40 fruits for each plant type at a time.

### 4.8. qRT-PCR Analysis

The total RNA was extracted from different samples including flesh prior to harvest and the injection zone of transient overexpression using CTAB methods, after which 1 μg RNA was used for the first cDNA synthesis. For the relative expression level investigation, 20 µL of a PCR mixture including 5 µL ddH_2_O, 10 µL SYBR Green I master mix, 1 µL forward primer, 1 µL reverse primer, and 3 µL cDNA template were used for qRT-PCR. Real-time PCR reaction was run on a LightCycler^®^ 480 system with a 96-well plate accompanied by the PCR procedure as follows: 95 °C for 5 min, followed by 45 cycles of 10 s at 95 °C, 20 s at 60 °C, and 20 s at 72 °C. Kiwifruit *β-actin* gene was considered as the control for normalization [46]. The relative expression levels of the genes were estimated by the 2^−ΔΔCt^ method [47].

### 4.9. Statistics

The significant differences between different groups were evaluated by the Student’s *t*-test. Differences were considered statistically significant at *p* < 0.05. Figures containing bar and line graphs were prepared with GraphPad Prism 5 (GraphPad Software Inc., San Diego, CA, USA). 

## 5. Conclusions

In this study, we screened and identified a PG-encoding gene *AaPG18* through physiological detection combined with molecular investigation based on the transcriptomics approach. The subcellular localization showed AaPG18 was a membrane-localized protein that functioned in the outer layer of the cell. The heterologous and homologous overexpression in strawberry and kiwifruit confirmed the functional role of *AaPG18* in fruit ripening and softening processes. The current study will help researchers to further explore the intricate mechanisms underlying fruit ripening and softening and to develop cultivars with an extended shelf-life of fruits.

## Data Availability

Not applicable.

## Supplementary Materials

The following supporting information can be downloaded at: https://www.mdpi.com/article/10.3390/ijms23052597/s1.
