# Supplementary material for: Characterization and Identification of a Ripening-Related Gene AaPG18 in Actinidia arguta
Source: Int J Mol Sci. 2022 Feb 26;23(5):2597. doi: 10.3390/ijms23052597 (PMC8910643; doi:10.3390/ijms23052597)
Supplement: Supplementary file 1 [file ijms-23-02597-s001.zip › Supplementary figures.pdf]

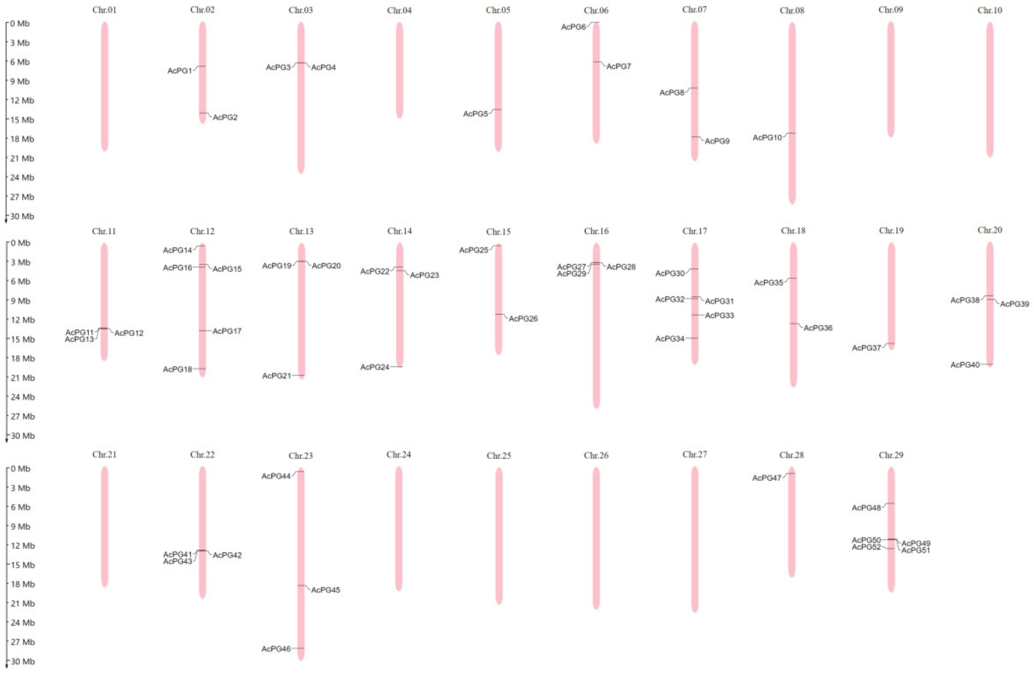

Figure S1. The chromosomal distribution of PG family members. 52 PG family members were irregularly distributed in 20 chromosomes including chromosome 02, 03, 05, 06, 07, 08, 11, 12, 13, 14, 15, 16, 17, 18, 19, 20, 22, 23, 28, 29. The black scale on the left represents the physical length of chromosome that is set up 30 Mb with the 3 Mb of interval. The gene locaters were marked as black solid lines on chromosomes. This figure was produced by an online tool MapGene2Chrom Web v2 (http://mg2c.iask.in/mg2c_v1.1/).

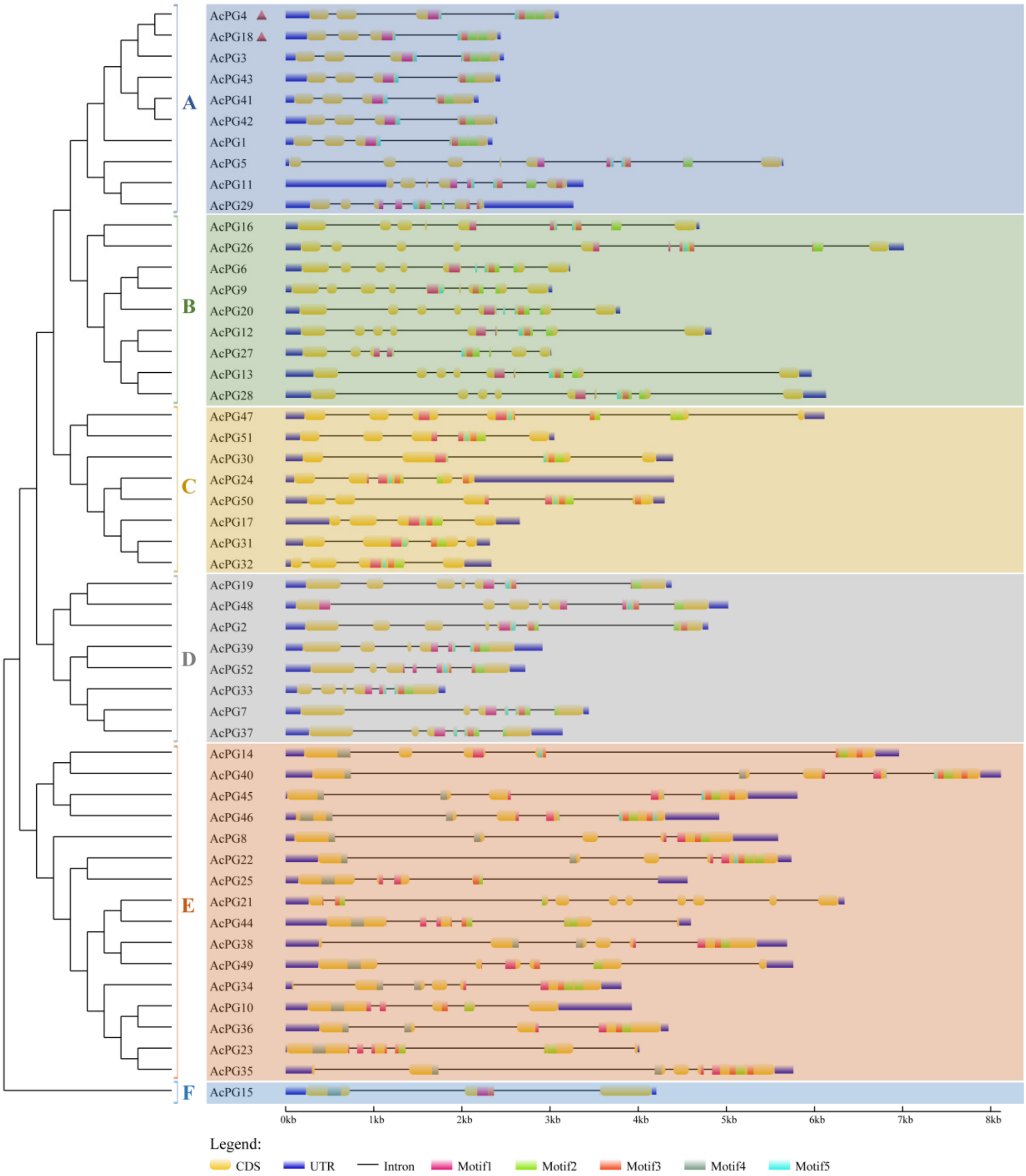

Figure S2. Phylogenetic evolution, domain organization and conserved motif analysis for PG family members. 52 PG members were divided into 6 sub-typed A, B, C, D, E and F. Exon and intron were indicated by the yellow box and black solid line, respectively. 5'- and 3'-untranslated regions (UTRs) were indicated by blue boxes. Five conserved motifs were represented by the pink, green, orange, blackish green and cerulean boxes at bottom. AaPG4 and AaPG18 were marked with red triangles. The specific details of multilevel consensus motifs were as follows:

Motif 1, APGDSPNTDGIHIDSSTNVVIEBSNIGTGDDCISIGSGWSN;

Motif 2, QGGGGYVRGITFQBITMENVKNPIIIDQNYCDHP;

Motif 3, VSNVTVENVTLTNTTNGVRIKTW;

Motif 4, LTGSFNLTSHMTLFLARDAVILGSQDEKEWPLIEPLPSYGRGRELPGGRY;

Motif 5, ITCGPGHGISIGSLG.
